# Risk assessment of the work-related musculoskeletal disorders based on individual characteristics using path analysis models

**DOI:** 10.1186/s12891-022-05573-6

**Published:** 2022-06-27

**Authors:** Ebrahim Darvishi, Fakhradin Ghasemi, Fateme Sadeghi, Kamaladdin Abedi, Somaye Rahmati, Ghazale Sadeghzade

**Affiliations:** 1 Environmental Health Research Center, Research Institute for Health Development, Kurdistan University of Medical Sciences, Sanandaj, Iran; 2 Department of occupational health and safety engineering, Abadan University of Medical Sciences, Abadan, Iran; 3 Department of Clinical Psychology, Faculty of Humanities, Kurdistan University, Sanandaj, Iran

**Keywords:** Musculoskeletal disorders, Risk assessment, Path analysis model, Individual characteristics

## Abstract

**Background:**

This study aimed to assess the risk of work-related musculoskeletal disorders (WMSDs) using the path analysis models.

**Methods:**

This study was carried out on 350 office employees with good general health. All variables were collected using a questionnaire. Personality traits and mental workload of employees were evaluated using the NEO Personality Inventory and the NASA-task load index software, respectively. The individual and personality traits were used as predictor variables, and mental workload (MWL) and body posture scores as mediating variables of the musculoskeletal discomforts. The role of predictor and mediating variables on discomforts was explained based on the path analysis models.

**Results:**

The impact coefficient of MWL and posture on WMSDs was significant. The coefficient of the direct effect of body mass index (BMI) and gender on musculoskeletal disorders was significant and positive and the women have reported a higher rate of discomforts. The strongest positive impact of personality traits on MWL and posture was conscientiousness, followed by neuroticism and agreeableness. In return, the strongest negative impact was extroversion, followed by openness. The strongest positive impact of individual factors on MWL and posture was BMI, followed by work experience.

**Conclusion:**

Gender, BMI, neuroticism, extraversion, and conscientiousness can be strong predictors for musculoskeletal discomforts which can mediate the impact of body posture and mental workload (mediating factors) on musculoskeletal discomfort. Therefore, personality and individual traits can be strong alarming and indicators for risk identification and preventing musculoskeletal disorders when choosing people for a job or task.

## Introduction

Work-related musculoskeletal discomforts (WMSDs) are common experiences in the workers involved in manual material handling and employees of official [1]. WMSDs arise in many tissues of the upper and lower limbs, neck, and lower back, such as muscles, tendons, joints, bones, ligaments, cartilage and intervertebral discs, peripheral nerves, and vascular system [2]. Despite the extent of mechanized processes, WMSDs are still the most common cause of time loss, compensation, and costs of work and occupational diseases. The mission of the NIOSH is to reduce the burden of WMSDs through a focused program of research and prevention, mitigate related risks, and improve the efficacy of workplace interventions [3].

Four categories of risk factors have been mentioned for the WMSDs [4]. There is a consensus that the main risk factors for WMSDs are the combination of individual and anthropometric factors (age, gender, BMI), genetic background [5], psychosocial (stress and mental workload), and biomechanical (posture, force exertion) [6]. The mechanical and occupational risk factors involve the force exertion to do tasks, the duration of the force employed, and the frequency at which tasks are done [7]. Although manual material handling is often a cause of acute injuries in workplaces, however, most of WMSDs are caused by repetitive movements or non-natural, static, awkward, and prolonged-time postures [8–11]. The psychosocial risk factors also involve job stress, high job requirements, mental workload, job dissatisfaction, and low social support. However, most studies have investigated the effect of biomechanics and occupational risk factors [8] and only a few have studied the relationship between psychological agents and the prevalence of WMSDs [2, 8, 12].

The relationship between personality traits and musculoskeletal diseases is not still entirely understood. Personality traits reflect people's thoughts, feelings, and behaviors [13]. The reactions of individuals may be different when exposed to workplace factors. According to psychology theories, personality traits can dictate adaptability to various conditions including work conditions. Theorists believe that personality traits are one of the factors that determine individual adaptation to the environment [14]. In the other words, personality is an active system within the individual composed of subjective experiences and mental aspects that get adjusted people to their surroundings [13]. Therefore, this system dictates differently in various individuals and reflects individual differences. One of the most practical determination methods of personality traits is the 5-factor neo-personality inventory, which is also widely used in job selection [15]. This tool investigates five traits of openness to experience, conscientiousness, extraversion, agreeableness, and neuroticism. Previous studies have shown that people with different personality traits perceive and react to events differently in certain conditions. Allread et al., have believed that using personality theory in the workplace helps us to better understand how people are injured while doing physical work [12]. Ghazanfari et al. have reported a significant relationship between personality traits and psychosomatic diseases [16]. Situation physical and mental of work to some, due to their personality type, maybe favorable and to others awkward. Thus, thoughts, feelings, and behaviors can affect other psychosocial risk factors in the workplace such as job stress, job dissatisfaction, and mental workload. On the other hand, the behaviors can determine patterns of occupational such as body postures during tasks. Bontrup et al. have reported that there is a strong association between sitting behavior and chronic LBP due to greater awareness of painless posture situations in people with chronic pain compared to people with acute pain [17].

Moreover, individual variables are person-specific attributes that have interaction with the other risk factors of musculoskeletal disorders. In other words, individual characteristics are a potential marker for development disorders and can effectively determine the WMSDs. However, many studies have confirmed that individual factors such as age, gender, body mass index, family history, genetics, etc., play an important role in developing WMSDs [18–20].

Although numerous studies have confirmed that mechanical risk factors such as awkward posture, force exertion, and repetitive movements, and psychological factors such as mental workload and personality traits and individual factors play a major role in the development of musculoskeletal disorders, however, none have examined the predictive and mediating role of musculoskeletal disorders risk factors. Since the individual and personality traits are identifiable, they can be used as predictive and alarming variables of musculoskeletal disorders. In other words, the risk of musculoskeletal disorders can be assessed and predicted using individual variables and personality traits. Therefore, using the data of the physical and mental risk factors in the task, we used structural equation modeling to examine if the big five personality traits help explain why posture and mental workload in certain individuals is awkward and extreme while others are not, even under nearly identical work conditions. Since the two main risk factors in the incidence of musculoskeletal disorders are mechanical (e.g., posture, force, and frequency) and psychological factors in the workplace (e.g., mental workload), therefore, it is hypothesized that individual and personality traits are involved in the development of musculoskeletal disorders with an effect on factors of mechanical and psychological. For this aim, the predictive role of personality traits and individual variables was investigated using pathway analysis models based on the following hypotheses:

H1: Body posture while doing tasks and mental workload as main risk factors are influenced by personality traits.

H2: Body posture while doing tasks and mental workload as main risk factors are influenced by individual characteristics such as age, sex, body mass index, and family history.

## Methods

This study was carried out among sedentary office workers. Since the working task strongly affects sitting behavior, therefore, the study population was selected from among employees that worked in office jobs with setting posture, distinctive working tasks, and involved cognitive performance. Inclusion criteria were non-smoking, alcohol, and drugs. Furthermore, employees of work shift; those who had neurological disorders, tumors or a history of surgery, sclerosis, trauma or any break in the bone, osteoporosis, systemic skeletal disorders, and any diseases same diabetes were excluded from the study. Finally, according to the literature review and 96% confidence interval, 60% frequency of disorders in the office workers, and 50% error, 350 employees were selected as the study population. Written consent was obtained from all employees. Ethics committees of Kurdistan University of medical sciences (no.IR.MUK.REC.1399.167) and the Ministry of Health and Medical Education Iran have approved the study. This study was conducted from July to December 2020. Each participant was assessed during one complete working shift at worksites. To investigate the relationships between daily sitting behavior (score of posture) and WMSDs, the score of posture for each subject undertaking daily office tasks was assessed in the workstations.

### Description of model variables

In this study, the main variables were age, gender, BMI, family history, and personality traits, as input variables. Posture and mental workload as mediated variables, and WMSDS as output variable was considered. All data required were collected using a questionnaire. At first, individual variables such as age, gender, family history, body mass index (BMI), and physical activity were collected from the workers. To assess the personality dimensions of participants, the revised NEO personality inventory was used [15]. It examines five personality traits including openness to experience, conscientiousness, extraversion, agreeableness, and neuroticism. Neuroticism indicates the degree to which a person is prepared to experience negative emotions and suffer from mental disorders such as depression and anxiety. Extraversion shows the degree of activeness, decisiveness, excitement, and courage more of a person in the face of isolation and passivity. Openness also describes the breadth, depth, complexity, and creativity of the mind and experience of the person towards the finite subjective. Agreeableness represents the social and prosocial orientation against hostile feedback to others. Some of these features may be depicted as altruism, kindness, trust, humility, etc. Conscientiousness describes people who perform their duties properly and have high planning power and have high effort and perseverance in performing their job duties. Then data were analyzed using a software version.

The mental workload (MWL) of employees was assessed using the NASA-Task Load index software as the received cognitive load [21]. MWL is a subjective and reflective estimation of the psychological load undergone due to work. NASA TLX is a weighted average of six scales demands of mental, physical, and Temporal, and level of Performance, Effort, and Frustration. This tool estimates workload using a 100-points scale. The final point is calculated based on the weighting average of six subscales and comparing them pairwise. (Table 1).

**Table 1:** Description of mental workload subscales in NASA task load index

Title	Descriptions	Endpoints
Mental Demand	Low/High	The required psychological and perceptual activity (e.g., thinking, calculating, remembering, deciding)
Physical Demand	Low/High	The required physical activity (e.g., pushing, pulling, swinging, handling, bending)
Temporal Demand	Low/High	The rate of time pressure felt during performing the task
Effort	Low/High	The level of effort demanded to achieve the optimal performance
Performance	Good/Poor	The Level of performance satisfaction in performing tasks
Frustration Level	Low/High	Feel insecure, discouraged, irritated, stressed, and annoyed versus secure, gratified, relaxed, and complacent during the task

### Determination of musculoskeletal discomforts

The musculoskeletal discomforts were assessed using the Cornell questionnaire. This tool was designed by Hedge et al. at the Cornell University and developed [22]. It contains a diagram of the body and 54-item questions about the frequency and intensity rate and the level of interference of musculoskeletal discomforts with the ability to work in 20 of the body regions during the last workweek. The musculoskeletal discomfort score in each part of the body is calculated according to the frequency, severity rate, and interference rating of discomfort. This tool measures generally the frequency and intensity rate of musculoskeletal pains in 12 parts of the body including the neck, right and left shoulder, upper back, right and left upper arm, lower back, right and left forearm, right and left wrist, hip, right and left thigh, right and left knee, right and left lower leg, and right and left feet respectively. The musculoskeletal discomforts score was acquired by summing by multiplying the frequency score (0, 1.5, 3.5, 5, and 10) by the severity rate (1, 2, and 3) for each body part. To compute data, missing values for the frequency or severity, zero is considered.

## Determination of body postures score

The posture of office workers may be restricted by construction and regulation of work stand dimensions, and the height of the desk and seat with additional screen adjustment maybe impose on workers a specific posture. Therefore, in this study, just office workers with similar workstation conditions (mainly desk and similar office adjustable chairs and also the same monitors, mouse, and keyboards) were evaluated. Because this study intended to identify postural stress (inefficient posture, repetitive movements, or prolonged time in the same positions) as a result of attitude, habits and personal characteristics, and inner tendencies. As a result, the body posture score was evaluated using the method of Rapid Office Strain Assessment (ROSA). ROSA is a picture-based posture checklist designed to quantify the risks work related to the postures from the computer user's workstation and work environment of office workers. This ergonomic assessment tool had been designed in a worksheet including four sections of score charts. Therefore, the user must observe the postures from the computer user's workstation and work out the components of the ROSA scoresheet. The sub-sections include seat height and chair pan, telephone and monitor, backrest and arm support, and keyboard and mouse. The ROSA final score is obtained by summing the scores recorded in each sub-section. The final score is indicative of the overall risk of musculoskeletal discomfort, as a result of personal habits and characteristics. Because the configuration of the office of all the workers was identical. The ROSA final scoring ranges from 1 to 10, with the higher score indicating an increased risk for work-related musculoskeletal disorders. Scores of greater than 5 are deemed to be “high risk” and the workstation should be assessed further.

## Development of path analysis models

In this study, four path analysis models (PAM) were developed to explore the role of individual and personality traits on WMSDs (target variable). To this end, the SPSS Amos version 19 was used.

PAM is a valuable method for evaluating the direct and indirect paths of independent variables on a specific target (dependent) variable. Each path has a coefficient in the range from − 1 to + 1 that equals standardized partial regression coefficients. A higher coefficient indicates that the variable has a higher effect on another. The significance of a path is determined using the t value that is the ratio of the unstandardized to standard error. If the value of t is more than 1.96, the path is significant at 0.05; if the value of t is more than 2.56, the path is significant at 0.01. To determine the fit goodness in a PAM, it can be used of indices available. Indices of absolute fit and comparative fit are two main groups of indices. The absolute fit indices are used to determine the fit of a hypothesized model with data. Some indices of this group are the model X^2^ values, goodness-of-fit index (GFI), and root mean square error of approximation (RMSEA), RMSEA has sensitive and instructive and easy-to-interpret nature. RMSEA index is computed as outlined below:

$$RMSEA=\sqrt{\frac{{X}^{2}-df}{df\left(N-1\right)}}$$

where X^2^ = model X^2^ value and N = sample size. If the RMSEA value is < 0.07 the fit model is good, values < 0.1 represent a mediocre fit, and values > 0.1 are unacceptable fit. It has been proposed that the model’s X^2^ value should be calculated using the ratio of the X^2^ to the df. In this state, a ratio < 2 is represented as a satisfying model fit. Indices of comparative fit demonstrate how close the hypothesized model is to a baseline ideal model. Normed fit index (NFI) and comparative fit index (CFI) are two examples of such fit indices. Moreover, comparative fit indices with values higher than 0.95 indicate that a model is of good fit.

## Results

The descriptive statistics of quantitative variables for the development of models was presented in Table 2. It shows that the highest score of mental workloads was the effort subscale and after that performance and mental demands, respectively. The overall average mental workload was estimated at 64.03 ± 24.98. Moreover, the score of posture represents that the level of WMSDs risk is medium.

**Table 2:** Descriptive statistics of quantitative variables for development of models

Quantitative variables	Minimum	Maximum	Mean	SD
Age (year)	18	62	34.68	8.25
Body Mass Index (kg/m^2^)	19.19	31.98	25.36	3.48
Body posture (score)	3	8	6.74	1.62
Mental demand (score)	5	100	76.31	22.00
Physical demand (score)	5	100	59.17	28.00
Temporal demand (score)	5	100	68.78	23.45
Effort (score)	5	100	77.81	19.67
Frustration (score)	5	100	50.85	28.92
Performance (score)	5	100	77.31	20.02
Mental workload (score)	16	100	64.03	24.98
Neuroticism (score)	27	80	51.69	8.96
Extraversion (score)	14	85	51.47	11.61
Openness (score)	26	76	51.80	8.71
Agreeableness (score)	21	68	45.75	8.51
Conscientiousness(score)	24	69	47.21	11.58

**Fig. 1:**
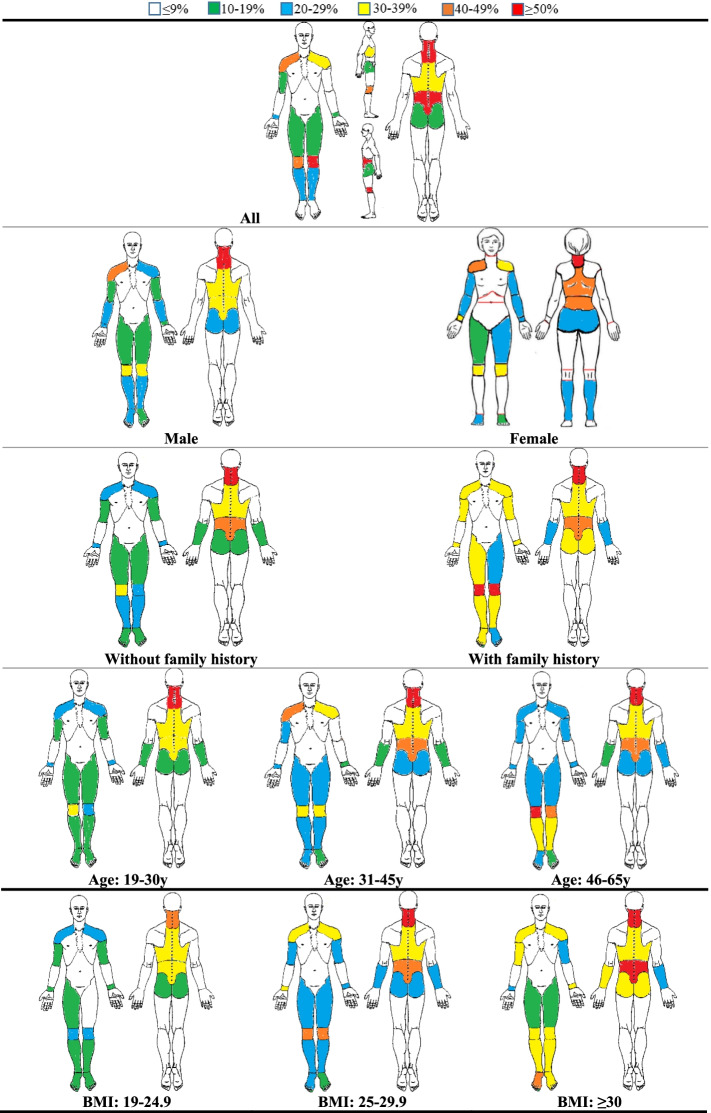
Percentage of the prevalence of musculoskeletal discomforts in body map (frontal and back) based on the individual variables (FH: family history)

**Fig. 2:**
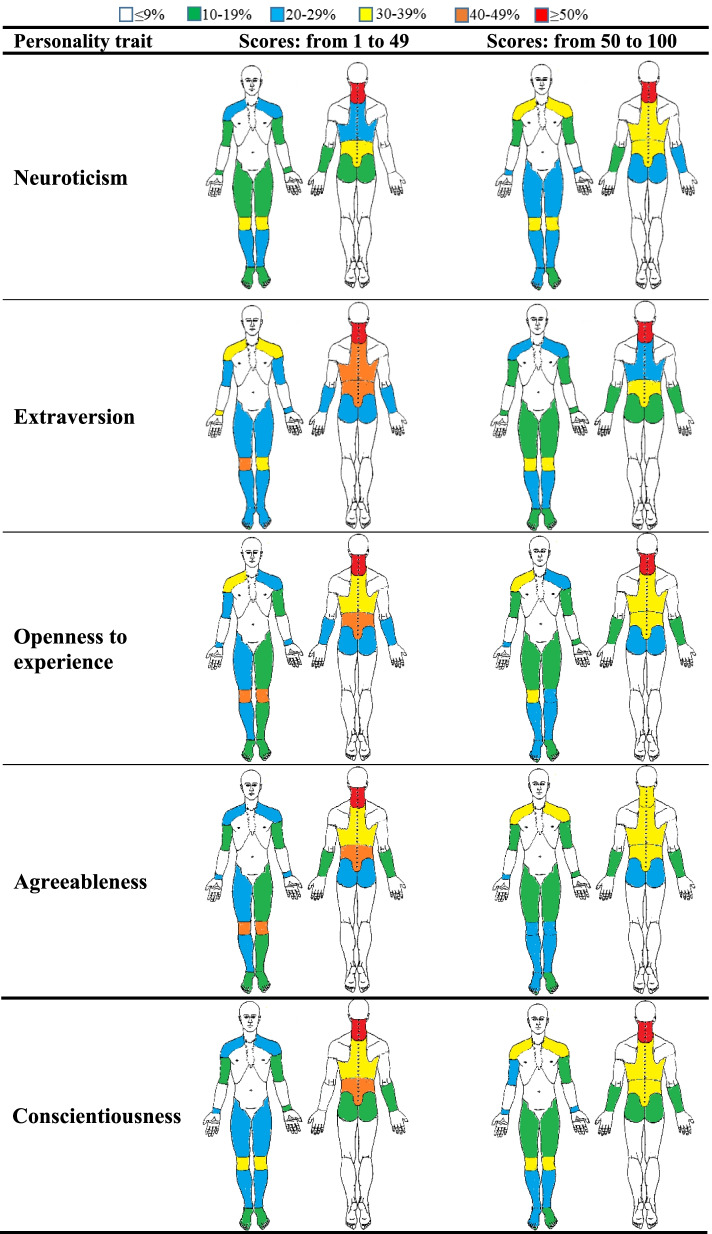
Percentage of the prevalence of musculoskeletal discomforts in body map (frontal and back) based on the personality traits

The descriptive statistics of WMSDs also was presented in Table 3. As shown, the prevalence of discomforts in the neck (18.30%), low back (16.50%), right shoulder (11.86%), and left and right knee (10.97% and 9.43%) respectively were reported more than in other body regions. Also, Figs. 1 and 2 show the percentage of the prevalence of musculoskeletal discomforts in body map based on the individual variables and personality traits, respectively.

**Table 3:** Descriptive statistics of the musculoskeletal discomforts’ prevalence

Part of the body	Frequency	Discomfort score	Prevalence (%)
Neck	11.31	12.48	18.30
Right Shoulder	06.96	8.09	11.86
Left Shoulder	06.14	3.24	4.75
Upper Back	05.69	3.11	4.56
Right Upper Arm	03.07	0.09	0.13
Left Upper Arm	02.76	0.09	0.13
Lower Back	09.90	11.25	16.50
Right Lower Arm	02.71	0.10	0.14
Left Lower Arm	02.41	0.10	0.14
Right Wrist	04.26	2.42	3.55
Left Wrist	03.29	0.72	1.05
Hip	03.87	3.64	5.34
Right Thigh	03.65	1.34	1.96
Left Thigh	03.35	3.41	5.00
Right Knee	06.76	6.43	9.43
Left Knee	07.33	7.48	10.97
Right Upper Feet	04.55	0.54	0.79
Left Lower Feet	04.63	1.72	2.52
Right Feet	02.55	1.20	1.76
Left Feet	01.64	0.72	1.05

**Fig. 3:**
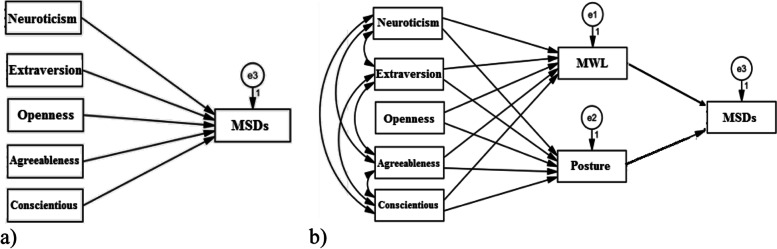
The models of describing the path of personality traits on musculoskeletal disorders (MSDs) a) the direct path, b) the indirect path (mediating role of mental workload (MWL) and body posture)

**Fig. 4:**
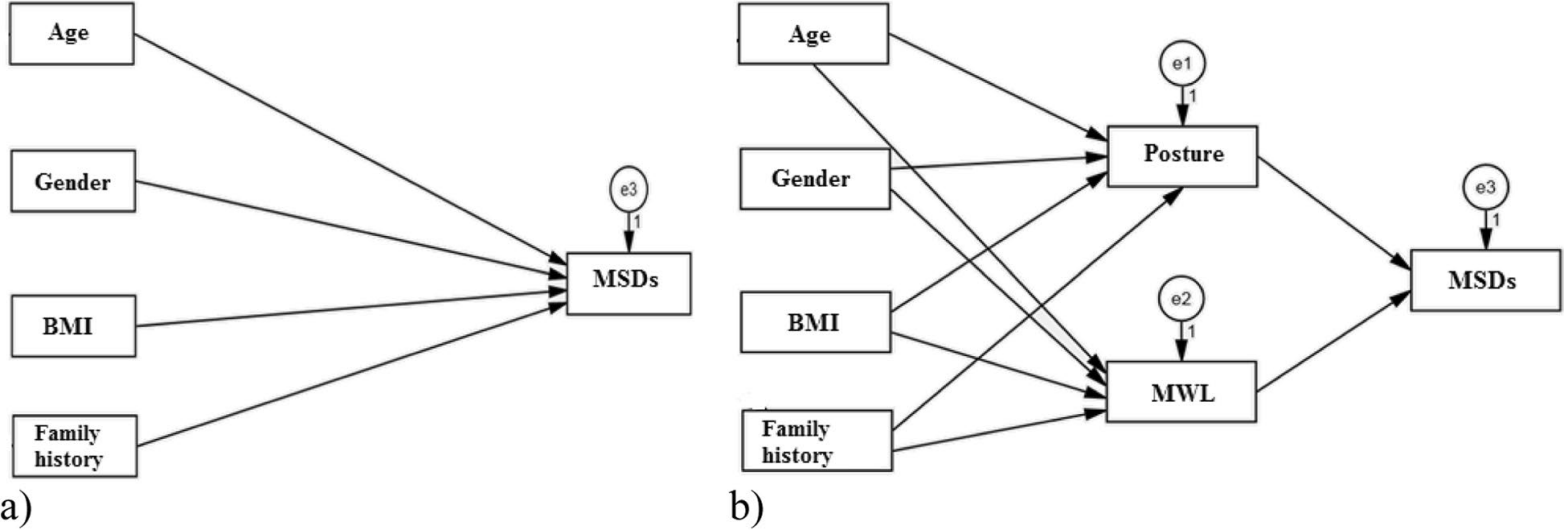
The models of describing the path of individual variables on musculoskeletal disorders (MSDs) a) the direct path, b) the indirect path (mediating role of mental workload (MWL) and body posture)

Based on the assumptions of the study about the effect of personality traits and individual variables on WMSDs, two models (one for personality traits and one for individual factors) were constructed, therefore, as shown in Figs. 3 and 4, to develop the models with acceptable fit, the paths were depicted. The most favorable fit indices of CFAM were used for the personality traits and WMSDs in the present study (Table 4). As shown, all the values of fit indices of the model were acceptable. According to the fit indices, the models were developed.

**Table 4:** Fit indices of the confirmatory factor analysis models for personality traits and musculoskeletal discomforts

Model fit index	Values		Acceptable level
	**Indirect model**	**Direct model**	
**Chi-square/df**	3.0	2.64	˂3
**RMSEA**	0.077	0.069	˂0.08
**IFI**	0.961	0.971	˃ 0.9
**NFI**	0.943	0.954	˃ 0.9
**GFI**	0.979	0.988	˃ 0.9
**CFI**	0.959	0.970	˃ 0.9
**TLI**	0.943	0.909	˃ 0.9

Note: *RMSEA* Root Mean Square Error of Approximation, *IFI* Incremental fit Index, *NFI* Normed Fit Index, *GFI* Goodness-Of-fit Index, *CFI* Comparative fit Index, *TLI* Tucker-Lewis’s index

Figure 3 illustrates the models explaining the path of personality traits on musculoskeletal discomforts directly and indirectly (the mediated role of mental workload (MWL) and posture).

The results of all information related to each path, from personality traits to MSDs, directly and indirectly, and the significance level of each path are presented in Table 5. As can be seen, the path coefficient of conscientiousness, neuroticism, and agreeableness on MWL and posture was positively followed respectively. In return, the path coefficient of extroversion and openness were followed negatively. Also, the path coefficient of MWL and posture on MSDs was significant.

**Table 5:** Regression weights and path coefficients of personality traits on musculoskeletal disorders

Path			Unstandardized path coefficient	Standardized path coefficient	SE	CR	*P*-value
**From**		**To**					
Neuroticism	–-˃	MWL	0.273	0.019	0.142	1.923	**0.054**
Extraversion	–-˃	MWL	-0.607	-0.282	0.128	-4.756	**˂0.001**
Openness	–-˃	MWL	-0.168	-0.059	0.146	-1.149	0.250
Agreeableness	–-˃	MWL	0.019	0.006	0.167	0.112	0.911
Conscientiousness	–-˃	MWL	0.233	0.008	0.110	2.124	**0.034**
Neuroticism	–-˃	Posture	0.019	0.102	0.012	1.604	0.109
Extraversion	–-˃	Posture	-0.030	-0.216	0.008	-3.585	**˂0.001**
Openness	–-˃	Posture	0.001	0.006	0.010	0.109	0.913
Agreeableness	–-˃	Posture	0.012	0.064	0.011	1.101	0.271
Conscientiousness	–-˃	Posture	0.023	0.397	0.007	3.220	**0.001**
MWL	–-˃	MSDs	1.596	0.261	0.260	6.131	**˂0.001**
Posture	–-˃	MSDs	49.98	0.532	3.999	12.50	**˂0.001**
Neuroticism	–-˃	MSDs	2.998	0.167	1.123	2.670	**0.008**
Extraversion	–-˃	MSDs	-2.228	-0.164	0.810	-2.750	**0.006**
Openness	–-˃	MSDs	-0.769	-0.042	0.937	-0.820	0.412
Agreeableness	–-˃	MSDs	0.826	0.045	1.060	0.779	0.436
Conscientiousness	–-˃	MSDs	1.866	0.134	0.842	2.216	**0.027**

*MWL* Mental workload, *MSDs* Musculoskeletal discomforts

There was a significant difference between some of the personality traits and the MWL directly. As summarized in Table 5, There was a positive path coefficient and significance between neuroticism and conscientiousness and MSDs. in return, the path coefficient of extraversion on MSDs was negative and significant. Table 6 shows the fit indices for models describing the path of individual variables on MSDs.

**Table 6:** Fit indices of the confirmatory factor analysis models for individual variables and MSDs

Model fit index	Values		Acceptable level
	**Indirect model**	**Direct model**	
**Chi-square/df**	6.347	5.315	˂3
**RMSEA**	0.124	0.111	˂0.08
**IFI**	0.812	0.883	˃ 0.9
**NFI**	0.785	0.860	˃ 0.9
**GFI**	0.965	0.970	˃ 0.9
**CFI**	0.805	0.880	˃ 0.9
**TLI**	0.590	0.760	˃ 0.9

Note: *RMSEA* Root Mean Square Error of Approximation, *IFI* Incremental fit Index, *NFI* Normed Fit Index, *GFI* Goodness-Of-fit Index, *CFI* Comparative fit Index, *TLI* Tucker-Lewis’s index

Figure 4 also illustrates the models of explaining the path of individual variables on musculoskeletal disorders directly and indirectly (the mediated role of mental workload (MWL) and body posture).

Table 7 represents the results of all information related to each path from individual variables to MSDs, directly and indirectly, and the significance level of each path. As shown, the path coefficient of BMI and work experience on MWL and posture was followed positively. Also, there was a significant difference between BMI and gender on MSDs directly.

**Table 7:** Regression weights and path coefficients of individual factors on musculoskeletal disorders

Path			Unstandardized path coefficient	Standardized path coefficient	SE	CR	*P*-value
**From**		**To**					
Age	–-˃	Posture	0.005	0.025	0.017	0.298	0.765
Gender	–-˃	Posture	0.032	0.009	0.202	0.157	0.875
BMI	–-˃	Posture	0.097	0.215	0.024	4.085	**˂0.001**
FH	–-˃	Posture	0.041	0.192	0.019	2.156	**0.031**
Age	–-˃	MWL	0.265	0.086	0.277	0.955	0.339
Gender	–-˃	MWL	-2.195	-0.039	3.039	-0.722	0.470
BMI	–-˃	MWL	1.929	0.279	0.422	4.577	**˂0.001**
WE	–-˃	MWL	-0.204	-0.062	0.304	-0.673	0.501
Posture	–-˃	MSDs	42.213	0.637	4.507	9.366	**˂0.001**
MWL	–-˃	MSDs	1.328	0.307	0.201	6.605	**˂0.001**
Age	–-˃	MSDs	0.689	0.035	1.703	0.405	0.686
Gender	–-˃	MSDs	56.617	0.158	21.902	2.585	**0.010**
BMI	–-˃	MSDs	13.276	0.288	3.838	3.459	**˂0.001**
WE	–-˃	MSDs	2.422	0.114	1.799	1.346	0.178

*FH* Family history, *MWL* Mental workload, *BMI* Body mass index

## Discussion

This study intended to predict the role of individual and personality traits in musculoskeletal discomforts among office employees. In general, musculoskeletal discomforts were very common among office employees. The results indicated that the most prevalent musculoskeletal discomforts among the studied employees were related to the neck, low back, right shoulder, knees, and hip, respectively. The high prevalence of discomfort particularly in the neck, low back, and hip are probably due to continuing sitting position and the lack of adequate exercise among employees. In general, the low back pain and other MSDs have a high prevalence among office employees, and in the meantime, the individual variables and personality traits are considered as one the potential risk factors [14, 23, 24].

The studies indicate that awful posture, force exertion, repetitive movement, and prolonged sitting are the main risk factors for MSDs among office workers. Therefore, the overall risk of musculoskeletal discomfort was estimated using the ROSA method. Given the same workstation and office configuration and the identical work tasks, the ROSA final score provided the level of change based on the risk associated with the person's mental feedback. Furthermore, assessment results of mental workload among office workers showed that the total score of mental workload among employees was more than the permissible limit. Numerous studies have confirmed that the rate of mental workload is high among office workers [25, 26].

Although many studies have proven that awkward posture, mental workload, and job stress are major risk factors for musculoskeletal disorders, however considering the role of personality traits in work-related musculoskeletal disorders is an issue that has not been identified. Therefore, based on previous studies, the hypothesis was formed that by using individual characteristics and personality traits, it is possible to identify people predisposed to musculoskeletal disorders. Thus, the role of the predictor of individual variables and personality traits on musculoskeletal discomforts directly and indirectly (considering body posture and mental workload as mediating variables) was analyzed using pathway analysis models. The ​results of the developed models indicated that several individual variables and personality traits can affect MSDs indirectly and directly. Some of them can be mediated or moderated the MSDs.

The results of the present study showed that personality traits both directly and indirectly can affect MSDs. In the indirect effect of personality traits on musculoskeletal discomforts, posture is influenced by personality traits and can be a mediator. Hence, the neurotic, introverted, and conscientious individuals have poor and improper physical positions and so experience awkward postures. In this regard, several studies have reported that postural stress and sitting behaviors for a prolonged time are influenced by individual characteristics [27]. Moreover, Govindu & Babski-Reeves have confirmed that psychosocial factors can affect changes in the position, movements, and forces exertion [28]. Calderwood et al. also have stated that personality traits affect individual fatigue levels [29]. Furthermore, the mental workload is influenced by personality traits and can be a mediator. Neurotic, introverted, and conscientious individuals experience more mental workload than those who are not. As noted, the MWL of employees was mainly due to three subscales of effort, performance, and mental demand, respectively. According to the finding of this study, the mental workload is more common in neurotic people than in non-neurotic people. Introvert individuals experience a greater mental load compared to extrovert individuals. The role of personality traits on MWL has rarely been investigated. Golmohammadi et al. believe that neuroticism and openness have a decisive role in mental workload and the mental workload is more common in introverted people than an extrovert people [30].

Various studies have also confirmed that neuroticism is associated with health problems [31, 32]. In this regard, many studies have highlighted that there is a significant positive relationship between the MWL and MSDs. Individuals during ongoing work and doing cognitive performance often pay less attention to their physique and posture. In this regard, Ghazanfari et al. have suggested that there is a significant positive association between neuroticism and openness and a significant negative association between extroversion and agreeableness with psychosomatic complaints [16]. In a cross-sectional study on 136 petrochemical workers, Ahmadi et al. have also concluded that personality traits especially extroversion and conscientiousness can contribute to musculoskeletal disorders [33].

The results of the present study also demonstrated that individual variables both directly and indirectly can affect MSDs. In the current study and under the developed models, people who had a higher BMI also had poorer body posture and a higher level of MWL than those who have not. The results also showed an awkward body posture in people with a family history of musculoskeletal disorders. The mental workload and working posture as mediating factors also directly affect MSDs. The direct pathway of BMI and family history on MSDs was also significant. The results of this study also indicated that gender has a significant path in musculoskeletal disorders and women experience a higher rate of disorders than men. Many studies have proven the role of individual factors such as gender, BMI, and family history on musculoskeletal disorders [9, 34]. Therefore, it is necessary to consider the personality traits and individual characteristics in the work system and organizational planning for preventing musculoskeletal disorders [18]. The developed pathway analysis models provided valuable information about how the direct and indirect effects of personality traits and individual variables on MSDs. Regardless of the direct effects of personality traits and individual variables on musculoskeletal disorders, according to the proposed models, body posture during tasks and the rate of mental workload experienced are influenced by individual and personality traits [35]. This means that we could be considered the role of the personality traits and individual variables in musculoskeletal disorders.

Although path analysis is a good approach for effects identification directly and indirectly, however, it has weaknesses. Path analysis is more explanatory than predictive. Therefore, for more accurate identification of risk factors, the use of neural network methods along with path analysis models will be very valuable. Another limitation is associated with cross-sectional studies. These studies often do not provide a precise basis for establishing causality. The current study was a questionnaire-based self-reported assessment; hence it may not reflect the perception of participants related to the pain and discomfort. Moreover, this study was conducted on office employees, which may not be representative of all employees. Future studies can be focused on risk assessment of musculoskeletal discomforts in workers by body posture of standing and manual material handling. However, this study does not downright imply that choosing employees with appropriate personalities and personal traits is the way to prevent WMSDs, but it points out that these factors could be good predictors of it.

## Conclusion

This study obtained important acumens into the predictive role of personality and individual characteristics in work-related musculoskeletal disorders. The results revealed that the risk factors for mechanical and occupational that develop musculoskeletal disorders are influenced by individual variables and personality traits. Family history, body mass index, and features such as neuroticism, introversion, and conscientiousness are strong predictors for work-related musculoskeletal disorders that modify the physical and mental workload, body posture, and other mechanical risk factors. Therefore, personality and individual traits could be considered for risk assessment and preventing musculoskeletal disorders when choosing people for a job or task. Since work-related musculoskeletal disorders are usually hardly curable, risk assessment and accurate prediction can be used by ergonomists to predict disorders and basic design of workstations.

## Data Availability

The datasets generated and/or analyzed during the current study are not publicly available due to limitations of ethical approval involving the patient data and anonymity but are available from the corresponding author on reasonable request.

## References

[1] Arvidsson I, Simonsen JG, Lindegård-andersson A, Björk J, Nordander C (2020). The impact of occupational and personal factors on musculoskeletal pain - a cohort study of female nurses, sonographers and teachers.

[2] Deng M, Wu F, Wang J, Sun L (2017). Musculoskeletal disorders, personality traits, psychological distress, and accident proneness of Chinese coal miners. Work.

[3] Mattila TEA, Perkiö-Mäkelä M, Hirvonen M, Kinnunen B, Väre M, Rautiainen RH (2022). Work exposures and mental and musculoskeletal symptoms in organic farming. Ergonomics.

[4] Sheikhzadeh A, Wertli MM, Weiner SS, Rasmussen-Barr E, Weiser S. Do psychological factors affect outcomes in musculoskeletal shoulder disorders? A systematic review. BMC Musculoskeletal Disorders. 2021;22. 10.1186/s12891-021-04359-6 PMC8214793 34147071

[5] Mehdizadeh A, Vinel A, Hu Q, Schall MC, Gallagher S, Sesek RF (2020). Job rotation and work-related musculoskeletal disorders: a fatigue-failure perspective. Ergonomics.

[6] Darvishi E, Maleki A, Giahi O, Akbarzadeh A (2016). Subjective Mental Workload and Its Correlation With Musculoskeletal Disorders in Bank Staff. J Manipulative Physiol Ther.

[7] Cabral AM, Moreira R de FC, Barros FC de, Sato T de O. Is physical capacity associated with the occurrence of musculoskeletal symptoms among office workers ? A cross ‑ sectional study. International Archives of Occupational and Environmental Health. 2019;92:1159–1172. 10.1007/s00420-019-01455-y 31273500

[8] Skov T, Borg V, 0rhede E. Psychosocial and physical risk factors for musculoskeletal disorders of the neck, shoulders, and lower back in salespeople. Occupational and Environmental Medicine. 1996;53:351–6. 10.1136/oem.53.5.351 PMC1128479 8673184

[9] Cole DC, Rivilis I (2004). Individual factors and musculoskeletal disorders: A framework for their consideration. J Electromyogr Kinesiol.

[10] da Silva JMN, da Silva LB, Gontijo LA (2017). Relationship between psychosocial factors and musculoskeletal disorders in footwear industry workers. Producao.

[11] Tirgar A, Khallaghi S, Taghipour M (2013). A study on musculoskeletal disorders and personal and occupational risk factors among surgeons. Iranian journal of health sciences.

[12] Allread WG. A study of the relationship between personality and risk factors for musculoskeletal disorders. In: Pmeeding.s of the IEA. 2000. p. 52–5.

[13] Kernberg OF (2016). What is personality?. J Pers Disord.

[14] Harkins SW, Price DD, Braith J (1989). Effects of extraversion and neuroticism on experimental pain, clinical pain, and illness behavior. Pain.

[15] RCosta RM, Paul C. The NEO Personality Inventory: Using the Five-Factor Model in counseling. Journal of counseling and development. 1991;69:367–72.

[16] Ghazanfari E, Feizi A, Fesharaki MG, Hassanzadeh A, Adibi P. The relationship between personality traits and psychosomatic complaints in a sample of Iranian adults. JOURNAL OF AFFECTIVE DISORDERS. 2019. 10.1016/j.jad.2019.10.020 31669924

[17] Bontrup C, Taylor WR, Fliesser M, Visscher R, Green T, Wippert PM, et al. Low back pain and its relationship with sitting behaviour among sedentary office workers. Applied Ergonomics. 2019;81 June:102894. 10.1016/j.apergo.2019.102894 31422243

[18] Piranveyseh P, Motamedzade M, Katerine Osatuke, Iraj Mohammadfam, Abbas Moghimbeigi AS& HM. Association between Psychosocial, Organizational and Personal Factors and Prevalence of Musculoskeletal Disorders in Office Workers. International Journal of Occupational Safety and Ergonomics. 2016;35:267–73. 10.1080/10803548.2015.1135568 26757785

[19] Soto-rodríguez FJ, Pérez-mármol JM, Muñoz-poblete C, Marzuca-nassr GN. The association of musculoskeletal complaints and individual and work-related factors with work ability in Chilean white- and blue-collar workers. International Journal of Occupational Safety and Ergonomics. 2020;0:1–23. 10.1080/10803548.2020.1865639 33331240

[20] Kaya G, Pt A, Pt TB, Pt ET. Musculoskeletal pain and its relation to individual and work-related factors : A cross-sectional study among Turkish office workers who work using computers. International Journal of Occupational Safety and Ergonomics. 2020;0:1–21. 10.1080/10803548.2020.1827528 32965164

[21] Cao A, Chintamani KK, Pandya AK, Ellis RD (2009). NASA TLX: Software for assessing subjective mental workload. Behav Res Methods.

[22] Çakıt : Erman. Ergonomic Risk Assessment using Cornell Musculoskeletal Discomfort Questionnaire in a Grocery Store. Ergonomics International Journal. 2019;3.

[23] Saklofske DH, Austin EJ, Rohr BA, Andrews JJW (2007). Personality, emotional intelligence and exercise. J Health Psychol.

[24] Fournier MA, Di Domenico SI, Weststrate NM, Quitasol MN, Dong M (2015). Toward a unified science of personality coherence. Can Psychol.

[25] Heidarimoghadam R, Saidnia H, Joudaki J, Mohammadi Y, Babamiri M (2019). Does mental workload can lead to musculoskeletal disorders in healthcare office workers? Suggest and investigate a path. Cogent Psychology.

[26] Darvishi E, Meimanatabadi M (2015). The Rate of Subjective Mental Workload and its Correlation with Musculoskeletal Disorders in Bank Staff in Kurdistan. Iran Procedia Manufacturing.

[27] Park SH, Lee PJ, Jeong JH (2018). Effects of noise sensitivity on psychophysiological responses to building noise. Build Environ.

[28] Beheshti MH, Taban E, Samaei SE, Faridan M, Khajehnasiri F, Khaveh LT, et al. The influence of personality traits and gender on noise annoyance in laboratory studies. Personality and Individual Differences. 2019;148 May:95–100.

[29] Calderwood C, Ackerman PL (2011). The relative impact of trait and temporal determinants of subjective fatigue. Personality Individ Differ.

[30] Golmohammadi R, Darvishi E, Shafiee Motlagh M, Faradmal J. Role of individual and personality traits in occupational noise-induced psychological effects. Applied Acoustics. 2021;173.

[31] Weinstein ND (1978). Individual differences in reactions to noise: A longitudinal study in a college dormitory. J Appl Psychol.

[32] Smith A (2003). The concept of noise sensitivity: Implications for noise control. Noise Health.

[33] Ahmadi O, Rasoulzadeh Y, Abbaspour A, Damanab poya shiekh, Rahimzadeh M, Keshizadeh F. Personality and Its Relationship with Prevalence of Musculoskeletal Disorders. jentashapir journal health research. 2017;9.

[34] Mehta RK, Agnew MJ (2016). Analysis of individual and occupational risk factors on task performance and biomechanical demands for a simulated drilling task. Int J Ind Ergon.

[35] Sanaeinasab H, Saffari M, Valipour F, Reza H, Mojtaba A (2018). The effectiveness of a model-based health education intervention to improve ergonomic posture in office computer workers : a randomized controlled trial. Int Arch Occup Environ Health.

