# Correction: Risk assessment of the work-related musculoskeletal disorders based on individual characteristics using path analysis models

**DOI:** 10.1186/s12891-022-06020-2

**Published:** 2022-12-01

**Authors:** Ebrahim Darvishi, Fakhradin Ghasemi, Fateme Sadeghi, Kamaladdin Abedi, Somaye Rahmati, Ghazale Sadeghzade

**Affiliations:** 1 Environmental Health Research Center, Research Institute for Health Development, Kurdistan University of Medical Sciences, Sanandaj, Iran; 2 Department of Occupational Health and Safety Engineering, Abadan University of Medical Sciences, Abadan, Iran; 3 Department of Clinical Psychology, Faculty of Humanities, Kurdistan University, Sanandaj, Iran

**Correction: BMC Musculoskelet Disord 23, 616 (2022)**

10.1186/s12891-022-05573-6

Following publication of the original article [1], the authors requested to insert funding institution “Research Deputy of Kurdistan University of Medical Sciences (Grant number: MUK.REC.1399.167)” under Funding section. The statement under Funding section should now read “This study was financially supported by the Research Deputy of Kurdistan University of Medical Sciences (Grant number: MUK.REC.1399.167).”
